# Mesenchymal Stromal Cell Bioreactor for *Ex Vivo* Reprogramming of Human Immune Cells

**DOI:** 10.1038/s41598-020-67039-w

**Published:** 2020-06-23

**Authors:** Ashley Allen, Natalie Vaninov, Matthew Li, Sunny Nguyen, Peter Igo, Arno W. Tilles, Brian O’Rourke, Brian L. K. Miller, Biju Parekkadan, Rita N. Barcia

**Affiliations:** 1 Sentien Biotechnologies, Inc., Lexington, MA 02421 USA; 2 Department of Surgery, Center for Surgery, Innovation, and Bioengineering, Massachusetts General Hospital, Harvard Medical School and Shriners Hospitals for Children, Boston, Massachusetts 02114 USA; 3 Harvard Stem Cell Institute, Cambridge, Massachusetts 02138 USA; 4 Department of Biomedical Engineering, Rutgers University, Piscataway, New Jersey 08854 USA

**Keywords:** Mesenchymal stem cells, Inflammation, Immunotherapy, Stem-cell biotechnology, Tissue engineering

## Abstract

Bone marrow mesenchymal stromal cells (MSCs) have been studied for decades as potent immunomodulators. Clinically, they have shown some promise but with limited success. Here, we report the ability of a scalable hollow fiber bioreactor to effectively maintain ideal MSC function as a single population while also being able to impart an immunoregulatory effect when cultured in tandem with an inflamed lymphocyte population. MSCs were seeded on the extraluminal side of hollow fibers within a bioreactor where they indirectly interact with immune cells flowing within the lumen of the fibers. MSCs showed a stable and predictable metabolite and secreted factor profile during several days of perfusion culture. Exposure of bioreactor-seeded MSCs to inflammatory stimuli reproducibly switched MSC secreted factor profiles and altered microvesicle composition. Furthermore, circulating, activated human peripheral blood mononuclear cells (PBMCs) were suppressed by MSC bioreactor culture confirmed by a durable change in their immunophenotype and function. This platform was useful to study a model of immobilized MSCs and circulating immune cells and showed that monocytes play an important role in MSC driven immunomodulation. This coculture technology can have broad implications for use in studying MSC-immune interactions under flow conditions as well as in the generation of *ex vivo* derived immune cellular therapeutics.

## Introduction

Immunotherapy can be broadly described as treatment of disease through the modulation of the immune system. Recently, cellular immunotherapy has drawn particular attention, especially in the field of oncology, as novel CAR-T therapies are being developed to elicit or amplify immune responses with tumor cytotoxicity as an end goal. But immunotherapy can also be deployed to broadly modulate the immune system and/or bring homeostasis to a dysregulated immune response. In complex conditions where single molecule immunotherapies have failed or cannot address broad, systemic pathways, cell therapy offers a compelling alternative approach.

Mesenchymal stromal cells (MSCs) have been described by many as potent immunomodulators capable of reprogramming, or differentiating, immune cells. Several studies have shown the differentiation of monocytes into regulatory macrophages^1–3^, dendritic and T cells into tolerance-inducing phenotypes^4^, and even naïve B cells into plasma cell types. MSCs represent a way to inhibit lymphocyte activation and reprogram the T and B cell sub-compartment through paracrine signaling mechanisms with a high degree of specificity. T cell activation is essential to an effective immune response, but prolonged T cell activation can lead to chronic inflammation and immunopathology. A skew towards a pro-inflammatory Th1 phenotype has been implicated in aberrant cell-mediated inflammation and autoimmune response in diseases such as type 1 diabetes, rheumatoid arthritis, multiple sclerosis, and systemic lupus erythematosus^5,6^. MSCs provide immunoregulatory assistance in order to quell this overactive Th1 profile and are a dynamic cell population that are apt to respond therapeutically to an inflamed cell population^7–9^. This potent effect is achieved through the MSC secretome, a complex mixture of bioactive factors and extracellular vesicles (EVs)^10^.

Clinical application of MSC technologies has been explored for some years and work is already underway. 2018 saw the first marketing approval of an allogeneic MSC product for the local treatment of complex perianal fistulas in Crohn’s disease^11^. In addition, systemic administration of allogeneic MSC treatment in pediatric patients with acute GvHD showed a significant improvement in survival rate and a sustained therapeutic effect at 6 months after treatment (NCT02336230). These recent events represent a revived success for MSCs, which have for decades delivered promising preclinical results without translation into significant clinical benefits. It is widely accepted that the root cause of failed clinical translation of systemic immunomodulation relates to issues with dosing and cell persistence^12^. Hours after conventional IV infusion, MSCs cannot be found in circulation^13–16^. In addition, different infused MSC products have been shown to display varying levels of procoagulant activity (via the expression of tissue factor) resulting in an instant innate immune attack that can compromise the safety and efficacy of the MSCs^17–19^. With these issues in mind, we have been developing an engineered approach to deliver MSC therapy, where the MSCs are inoculated in an ex vivo system, allowing the cells and the blood to communicate bi-directionally through distinct compartments^20^. This system enables greater visibility and control to the questions of persistence and dosing that have previously hampered clinical translation of MSC therapy.

This report describes a novel, scalable continuous flow MSC bioreactor that induces blood cell reprogramming. An advanced throughput bioreactor system was developed in which MSCs were seeded with stabilized function on the extraluminal surface of a membrane in basal perfusion media conditions. In the bioreactor configuration, MSCs were responsive to an inflammatory cytokine cocktail resulting in dynamic cytokine and growth factor secretion and increased microvesicle size. Exposure of bioreactor seeded MSCs to circulating immune cells in continuous flow led to the generation of an immune cell population with an altered surface phenotype, proliferation potential, and cytokine secretion profile. These results further elucidate the interplay between immune cells and MSCs and pave the way for novel MSC immunotherapy modalities.

## Results

### Metabolism and secreted factor release of immobilized MSCs in a parallel-throughput microreactor perfusion system

Human MSCs were obtained from the bone marrow of healthy donors. Cells (P3-P5) were characterized as MSC through immunophenotyping of surface markers with flow cytometry. The expression rates of the MSC markers CD73, CD90, CD105, and CD166 were 99.6%, 99.9%, 99.8% and 99.2% positive and negative MSC markers CD14, CD34, and CD45 were 2.3%, 0.8% and 1.1%, respectively.

We developed a process to seed and perfuse MSCs in a continuous flow bioreactor within a recirculating fluid handling system (Supplemental Fig. 1A). Current in vitro co-culture platforms, namely transwells, prevent the longitudinal study of MSC:PBMC doses above a certain threshold without encountering challenges associated with available surface area and nutrients^3,21^. To circumvent this problem, we used hollow-fiber microreactors with a semi-permeable membrane (0.2 μm pore size) to characterize the performance of MSCs in vitro. MSC bioreactors were first perfused with media to characterize MSC secreted factors, which will be referred to as the cells’ pharmacokinetics (PK). Bioreactors were seeded at various cell densities and incubated to allow cells to attach. After attachment, devices were integrated into cell perfusion circuits. Multichannel peristaltic pumps allowed for a medium throughput assessment of microreactor performance as 8 devices could be perfused in parallel with each pump (Supplemental Fig. 1B).

**Figure 1:**
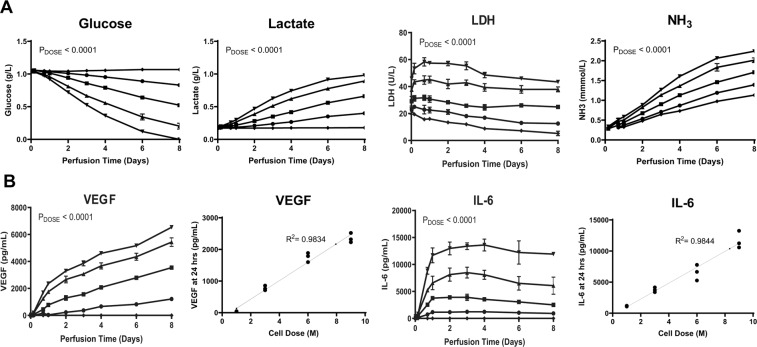
Dose-dependent MSC Metabolism and Measurement of Secreted Factors During 8-Day Microreactor Continuous Perfusion. Microreactors seeded with 0 (◆), 1 ×10^6^ (∙), 3 ×10^6^ (▪), 6 ×10^6^ (▴), and 9 ×10^6^ (▼) viable MSCs were incubated for 2 hours at 37 °C followed by a 24 hour hold at room temperature. Microreactors were then perfused with media containing 10% FBS, 1% antibiotic-antimycotic, 0.2% gentamicin and sampled over 8 days. **(A)** Glucose (g/L), Lactate (g/L), LDH (U/L), and NH_3_ (mmol/L) were quantified via CedEx Bio measurement on thawed samples (**A**). Measurement of secreted factors in these samples was performed via ELISA for IL-6 (pg/mL) and VEGF (pg/mL) (**B**). Graphs show average values for each cell dose group + standard deviations for each. Two-way ANOVA was performed for all timepoints with n = 3 (Days 1-4) showing a significant impact of dose on device metabolism and secreted factor performance. Correlation of cell dose to device IL-6 and VEGF performance at 24 hours of perfusion is shown for individual devices.

To study system metabolites and PK, microreactors were seeded with 0, 3, 6, or 9 ×10^6^ viable MSCs per device and then perfused at 10 mL/min within the 50 mL circuit. 50 mL of complete media allows enough volume for longitudinal sampling and an excess of nutrients. Sampling was performed from 30 minutes to 8 days and analyzed for metabolites to assess functional growth biomarkers. Cellular metabolism of glucose was increased with cell dose (Fig. 1A). Metabolites showed a similar relationship, with correlations of dose to lactate and ammonia production at Day 1 showing a linear relationship (R^2^_Lactate_ = 0.994, R^2^_Ammonia_ = 0.988). Strong correlations (R^2^ > 0.95) were observed for other timepoints after 24 hours (data not shown). Glucose was consistently consumed and, for the highest MSC dose, depleted within the perfusion circuit by 8 days of perfusion. LDH showed a different trend, with values increasing from 30 minutes to 24 hours and then decreasing thereafter (presumably due to degradation), revealing that the majority of LDH production occurs initially and is linear with cell dose across timepoints (R^2^_LDH, 0.5hr_ = 0.9998, R^2^_LDH, 24hr_ = 0.9986). Taken together, these results show that MSCs are viable out to 8 days of perfusion and all cell doses tested behaved in a predictable way in terms of cellular metabolism during this perfusion timeframe.

Measurement of prototypical MSC-secreted factors via ELISA analysis showed accumulation of both IL-6 and VEGF over time with dose-dependent output of secreted factors (Fig. 1B). Correlation of factor production to cell dose revealed linear scaling for both IL-6 and VEGF(Fig. 1A,B). The highest rate of secreted factor production occurred within the first 24 hours of perfusion (Supplementary Figure 2). After this, IL-6 accumulation continued to increase and then peaked at 72 hours of perfusion with measured IL-6 decreasing slightly over time thereafter. VEGF established a relatively fixed rate of output for each dose over the first 2 days and concentration was thereafter sustained out to 8 days of perfusion. These results indicate that while each factor may have a unique PK profile longitudinally, discrete doses of MSCs result in a reproducible and dose-dependent PK response at basal levels.

### Steady-state and dynamic secreted factor response of MSCs in continuous perfusion

**Figure 2:**
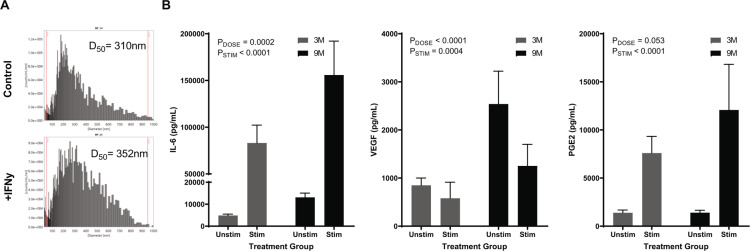
Dynamic Modulation of MSC Bioreactor Output with Introduction of Inflammatory Stimulus. Microreactors were seeded and perfused in a 50-mL circuit containing inflammatory stimuli. Particle Size Analysis of MSC extracellular vesicles (MSC-EVs) produced by bioreactors seeded with 3 ×10^6^ cells after 12 hours of perfusion with or without IFNγ stimulation (5 ng/mL) (**A**). Secreted factors (IL-6, VEGF, and PGE2) were measured via ELISA after seeding microreactors with 3 ×10^6^ (gray) or 9 ×10^6^ (black) viable MSCs, incubating for two hours at 37 °C, hold for 24 hours at RT, and perfusion with or without an inflammatory cocktail (10 ng/mL IFNγ, 1 ng/mL IL-1β, and 5 ng/mL TNFα) for 48 hours (**B**). Graphs show average values for each cell dose group + standard deviations for each (n = 2 donors, n = 3/donor). Two Way ANOVA was performed on each set of samples to determine effects of dose (P_DOSE_) and stimulation (P_STIMULATION_) on MSC output.

Upon establishing steady-state dynamics, new microreactors were prepared and perfused under basal or inflammatory conditions. Inflammatory stimuli were added at the start of perfusion and samples were collected at 12, 24, and 48 hours. Mass spectroscopy particle size analysis of extracellular vesicles showed that at 12 hours, the milieu conditioned by MSCs in response to inflammatory stimulus had an increased average particle size in comparison to the unstimulated control (Fig. 2A, stimulated D_50_ = 310 vs. control D_50_ = 352). Ultracentrifugation of the media verified a concentrated sample for analyses that stained positively for membrane encapsulated particles that also contained miRNA content (Supplementary Figure 3). The average rate of exosome production was ~1600 particles/min/10^6^ cells. Samples taken at 48 hours after stimulation showed a measurable and sustained increase in production of secreted factors, IL-6 and PGE2, compared to unstimulated controls (Fig. 2B). This effect appeared to be dose-dependent in IL-6 (P_Dose,T24_ = 0.0001, P_Dose,T48_ = 0.0002), with all devices increasing in IL-6 output compared to unstimulated controls (Fold Change: 3M_T24_ = 20.04 + 7.1, 3M_T48_ = 17.0 + 3.3, 9M_T24_ = 16.6 + 5.7, 9M_T48_ = 11.9 + 2.2). PGE2 similarly showed an increase with stimulation (Fold Change: 3M_T24_ = 8.3 + 6.4, 3M_T48_ = 5.9 + 2.9, 9M_T24_ = 14.3 + 16.2, 9M_T48_ = 8.7 + 3.3), but the fold increase was less than what was observed for IL-6 and did not appear to be dose-dependent. Interestingly, VEGF showed no effect of stimulation on output at 24 hours followed by decreased production at 48 hours compared to unstimulated controls (Fold Change: 3M_T24_ = 2.5 + 3.2, 3M_T48_ = 0.72 + 0.46, 9M_T24_ = 0.50 + 0.14, 9M_T48_ = 0.54 + 0.14). Metabolite data showed that cell metabolism in stimulated devices did not change compared to control (data not shown). This stimulation data shows that cells in the bioreactor sense and respond to an inflammatory stimulus via differential secreted factor response.

### Bioreactor Treatment with MSCs Modifies PBMC Secreted Factor Milieu

**Figure 3:**
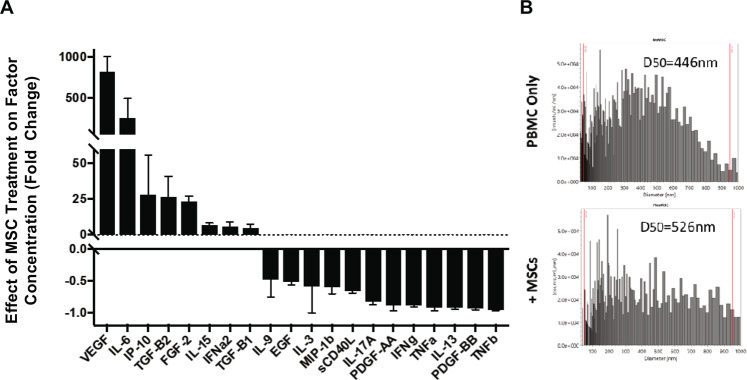
Secreted Factors following Perfusion of Activated PBMCs through MSC-Bioreactor Circuits. PBMCs activated with PHA (5 ug/mL) and supplemented with IL-2 (100 ng/mL) were perfused through bioreactor circuits containing 0 M or 9 M (n = 3) and medium was recovered. Both (**A**) factor concentration and (**B**) extracellular vesicle characteristics were modulated with MSC treatment.

Media spiked with activated human peripheral blood mononuclear cells (PBMCs) was next used to investigate bioreactor effects on immune responses, referred to as the bioreactor pharmacodynamics (PD). Platform specifications were altered for PBMC perfusion to maximize longitudinal viability of the cells in the perfusion circuit. To study bioreactor PD effects, reactors seeded with 0 M (acellular control) or 9 M MSCs were perfused with activated PBMCs over 5 days. Medium was then recovered from the circuits and analyzed for cytokine content to examine the impact of MSC treatment on secreted factor milieu (Fig. 3A). Many known MSC-factors were increased in circuits treated by MSC-seeded microreactors, including VEGF and IL-6. Additionally, several cytokines were decreased with MSC treatment. While the source of these factors cannot be definitively established, those attenuated in the presence of MSCs are likely produced by the PBMCs and/or consumed by MSCs (e.g. TNFα). Collectively, a switch in secreted immune signaling and growth factors were observed in the presence of an MSC bioreactor compared to an acellular control.

Beyond secreted factor concentration, the impact of MSC-seeded bioreactors on extracellular vesicle size was also examined (Fig. 3). Interestingly, average EV size from cultures consisting of a single cell type was increased for those containing only PBMCs in comparison to stimulated MSCs alone (D_50_ = 446 nm vs. 352 nm). However, for the MSC:PBMC co-culture system, EVs were further enlarged on average. These data suggest that the factor milieu can be modulated with MSC treatment both directly through cell-secreted factors as well as indirectly via altered EV characteristics.

### Donor-independent reduction of human lymphocyte activation by MSC reactors is associated with a decrease in CD8+ cytotoxic T cells

**Figure 4:**
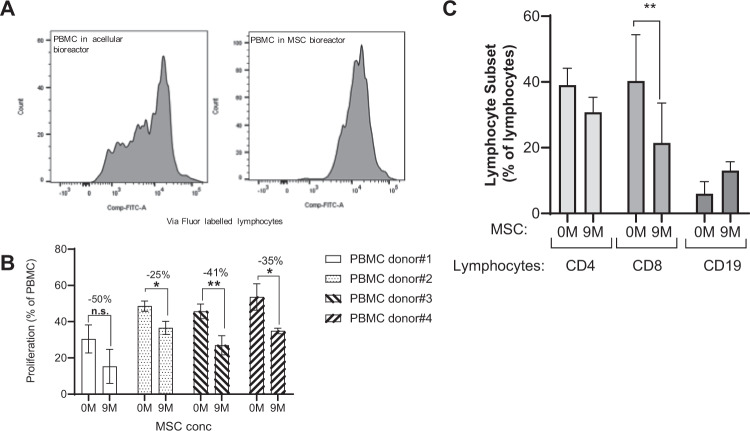
Prevention of lymphocyte activation in MSC bioreactors. PBMCs were labeled with ViaFluor, stimulated (PHA/IL-2) and perfused for 5-days via circuits containing microreactors seeded with 0, or 9 ×10^6^ MSCs per device. MSC treatment was shown to inhibit T-cell proliferation as demonstrated by ViaFluor labelling **(A)**. This was observed for four PBMC donors (n = 4 donors, n = 3/donor) **(B)**. Percent decrease in PBMC proliferation with MSC (9 M) is indicated. Perfusion via MSC-seeded microreactors (9 M) exhibited changes in CD4, CD8, and CD19 + lymphocytes when compared to acellular controls (0 M) (n = 2 donors, n = 3/donor) **(C)**. Graphs show average values for each cell dose group + standard deviations for two donors. A student’s t-test was performed on each set. **p ≤ 0.01 *p ≤ 0.05.

Previous work has shown that cell dose plays a key role in the ability of MSCs to modulate the immune system^22^. To explore whether MSCs had an immunomodulatory effect under dynamic flow conditions, PBMCs stimulated with PHA and IL-2 were perfused over 5 days through microreactor-containing circuits seeded with 0, 3 or 9 ×10^6^ MSCs. Using ViaFluor-labeled PBMCs, MSC-seeded (9 M) microreactors were shown to inhibit lymphocyte proliferation in comparison to acellular controls (0 M) for multiple PBMC donors (Fig. 4A). This observation was true for different PBMC donors (Fig. 4B). MSC treatment was further observed to impact the different lymphocyte populations. Total CD8 + T-cell population was decreased in MSC-containing circuits in comparison to acellular controls, while total CD4 + T-cells were marginally decreased. Conversely, MSC treatment enhanced CD19 + B-cells (Fig. 4C). Taken together, MSC treatment under dynamic flow appeared to have a significant and measurable immunomodulatory impact on PBMCs.

**Figure 5:**
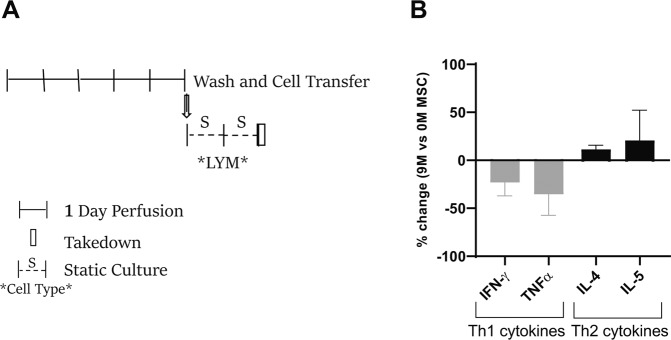
MSC bioreactor reprogramming of T cell cytokine responses. Stimulated (PHA/IL-2) PBMCs perfused in a bioreactor (+/− MSC) for 5-days were plated on a 6-well tissue culture dish for two additional days (**A**). Lymphocyte secreted factors (in the supernatant) were assayed using multiplex cytokine panel. Th1/Th2 specific cytokines are shown as a percentage change with MSC (9 M) treatment relative to acellular (0 M MSC) (n = 1 donor, 3 runs/donor) (**B**).

To test whether MSC exposure had a persistent reprogramming effect, MSC treated PBMCs were collected from the circuits following perfusion, resuspended in fresh media, and cultured for two additional days. Lymphocytes were then immunostained for chemokine receptors typically associated with Th1 (CXCR3) and Th2 (CXCR4) and assessed for cytokine production in post-perfusion culture (Fig. 5A). MSC treatment only slightly changed Th1/Th2 chemokine surface marker staining (data not shown). However, the 2 day post-perfusion system showed numerous increased (e.g. IL-4 and IL-5) and decreased (e.g. TNFα, and INFγ) factors suggesting a Th1-to-Th2 shift of the MSC-treated lymphocytes (Fig. 5B). These results also demonstrate that the reprogramming driven by the MSC bioreactor persists after the removal of the MSC bioreactor circuit.

**Figure 6:**
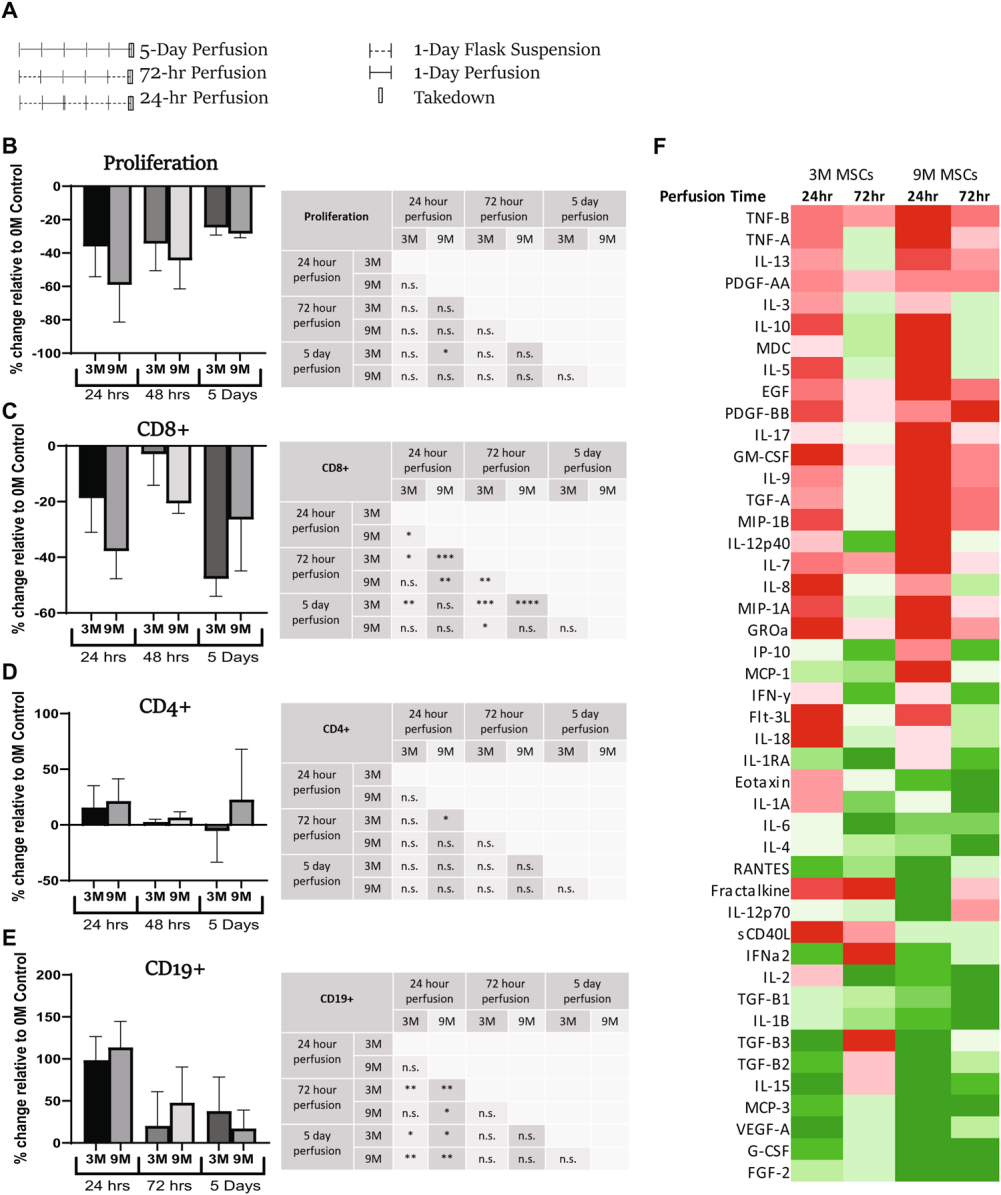
Dose and duration effects of *ex vivo* MSC perfusion on human lymphocytes (**A**) Stimulated (PHA/IL-2) PBMCs were perfused for either 24 hours, 72 hours or for 5 days through circuits containing microreactors seeded with either 0, 3, or 9 ×10^6^ MSCs per device (0 M, 3 M, 9 M) (n = 2 donors, n ≥ 3/donor) (5 day historical only has one donor). The 24- and 72-hour perfusion groups were first placed into static culture for 24 hours prior to perfusion initiation. Each group was perfused for the designated time and then placed into static culture until collection on Day 5. Relative to 0 M control MSC treatment was shown to inhibit lymphocyte proliferation in all conditions (**B**), with a trend correlating with MSC dose response. CD8 + T cell proliferation was also inhibited by perfusion (**C**) while B-cell proliferation increased (**E**) in a dose and duration dependent manner for each subpopulation. A student’s t-test was performed on each set. ****p ≤ 0.0001 ***p ≤ 0.001 **p ≤ 0.01 *p ≤ 0.05. n.s. = not significant. Graphs show average values for each cell dose + standard deviation. (**F**) Culture media samples were collected at Day 5 and analyzed via multiplex. Measurement of percent change was calculated by determining the output of any condition relative to the 0 M control. The absolute value of the percent change was then charted into columns according to MSC dose and perfusion duration. Comparative analysis of intensities were calculated within each row with darker colors representing larger values. Red blocks indicate decreases in percent change while green blocks indicate increases. Of all conditions, the 9 M MSC 24-hour perfusion group showed the largest changes in analyte values (n = 1 donor).

To further evaluate the persistence of the immunomodulatory effect observed as well as to characterize dosing, a study profiling PD outcomes of shorter perfusion durations (24-hr and 72-hr) across several MSC doses (0 M vs 3 M vs 9 M) was run (Fig. 6). The results of this study were compared to the historical 5-day perfusion study results. Between all conditions, 24 hours of perfusion with a 9 M MSC dose resulted in the strongest effect for all outcomes measured; lymphocyte proliferation (Fig. 6B) and CD8+ T-cell abundance decreased more strongly than CD4+ T cells (Fig. 6C,D), while CD19+ B-cell abundance increased most strongly relative to acellular controls (Fig. 6E). Likewise, between all groups tested, the greatest changes in inflammatory milieu were measured from the 24-hr perfusion group with 9 M MSCs (Fig. 6F). These results indicated that both the MSC dose and the duration of perfusion had a significant impact on immunoregulatory potential.

### Interplay between monocytes, MSCs and lymphocytes

**Figure 7:**
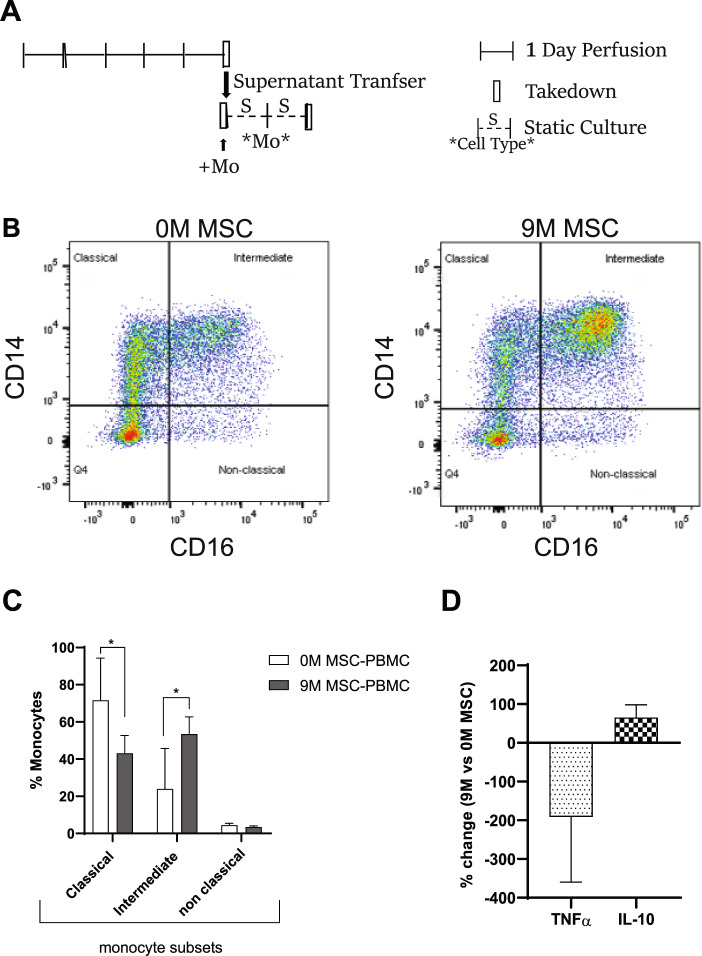
Transfer of MSC bioreactor/PBMC perfusate alter primary human monocyte differentiation. MSC reprogrammed PBMCs (PHA/IL-2 activated) perfusate/supernatant from a 5-day bioreactor (with −/+ MSC) was added on monocytes cultured on a cell-repellent tissue culture dish for two days (**A**). After two days, monocytes were stained using CD14 and CD16 antibodies and dot blots are shown (**B**) and percentage change in monocyte subsets with and without MSC-reprogrammed PBMC is shown (n = 2 donors, n = 3/donor) (**C**). The levels of TNFα and IL-10 in the supernatant of monocytes after two-days of addition of PBMCs perfusate from circuits with or without MSCs is plotted as percentage change with MSC addition (n = 2 donors, n = 3/donor) (**D**).

Since monocytes have a short half-life (1-2 days)^23^, they do not survive during a 5-day MSC-PBMC perfusion (data not shown). We therefore investigated the effect of MSCs on monocytes using two different approaches. The first approach was to assay the effect of MSC-reprogrammed PBMC perfusate/supernatant from a 5-day perfusion containing secreted factors onto statically cultured monocytes for two days (Fig. 7). This approach showed that the addition of MSC-PBMC supernatant induced changes in the monocyte subset population (Fig. 7B,C) by shifting the population from classical to intermediate monocytes. Interestingly, this was accompanied by a decrease in pro-inflammatory cytokine TNFα and an increase in anti-inflammatory IL-10 secretion compared to monocytes with addition of 0 M MSC-PBMC perfusate (Fig. 7D).

**Figure 8:**
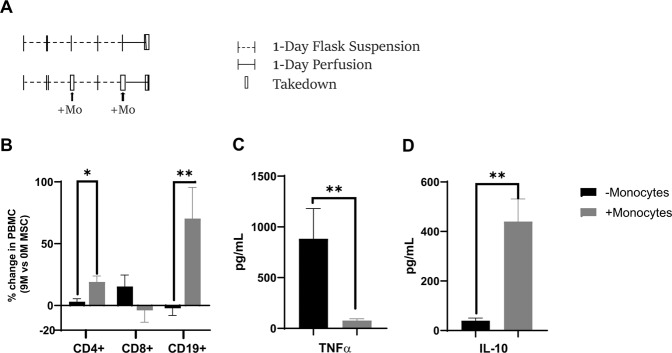
Role of monocytes in MSC bioreactor immune reprogramming. PHA/IL-2 stimulated PBMCs were cultured in static tissue culture flasks for 4-days. On day 2 and day 4, monocytes (~2.8 M/ bioreactor) were added into PBMC flask (in the monocyte addition group in gray). At day 4, after addition of monocytes PBMCs were perfused in bioreactor for 24 hours via circuits seeded with 0, or 9 ×10^6^ MSCs per device (**A**). Following 24-hours of perfusion, the cells were assayed via flow cytometry (**B**) and supernatant was assayed for TNFα and IL-10 levels both without and with monocyte addition (**C**). Overall, the addition of monocytes to the PBMCs enhanced the MSC immunomodulatory effects as shown by changes in lymphocyte population, TNFα and IL-10 levels (**D**). A student’s t-test was performed on each set (**p ≤ 0.01 *p ≤ 0.05). Graphs show average values + standard deviation (n = 1 donors, n = 3/donor).

To further understand the role of monocytes in MSC bioreactor immunomodulation, the second approach used a system where monocytes are naturally degraded over a 4-day static activation of PBMCs followed by 24 hours of MSC perfusion (Fig. 8A). In this setting, immune modulation is drastically reduced or absent (Fig. 8B). However, when we replenished monocytes by adding them back into the PBMC cultures at day-2 and day-4 prior to perfusion with MSCs, the immunomodulation was partly restored. Changes in CD4, CD8 and CD19 cells, similar to the ones observed with MSC immunomodulation (Fig. 4C), were observed when monocytes were added to the PBMC cultures prior to perfusion at day 4 (Fig. 8B). Furthermore, final TNFα levels were 11.47x lower when monocytes were added back to the PBMCs while IL-10 was increased 11.12 fold (Fig. 8C,D). This data is supportive of a critical role for circulating monocytes in MSC immunomodulation of lymphocytes.

## Discussion

Here we describe the development of a stable, long term, continuous perfusion platform that promotes the complex interactions between MSCs and immune cells. This platform can be utilized both for the in-depth study of such interactions as well as the development of a future ex vivo immunotherapy.

Endogenously, MSCs that regulate immune cells in the blood are adhered to the outside of microvessels and uptake factors from the inflammatory milieu, responding dynamically to the microenvironmental inflammatory cues around them^24,25^. MSC recognition of enriched factors from the inflammatory milieu results in MSC “priming” or “licensing” with an altered paracrine signaling^26,27^. Our engineered product, a hollow fiber bioreactor that houses the adherent MSCs, successfully reproduced these capacities. In this familiar structure and environment, MSCs were viable (Fig. 1), able to sense inflammatory signals and to dynamically respond with differential secreted factors and altered EV characteristics (Fig. 2). Due to the semi-permeable membrane with a cut-off of 0.2 um, there is likely skewing of the particle sizes of vesicles compared to a non-membrane sample collection. While quite intriguing, we acknowledge that the EV results herein reported are cursory and that greater depth delving into EV specific mechanisms of action are warranted in future studies.

In this controlled platform, MSCs were able to react and impart an immunomodulatory effect onto PBMCs under flow conditions, consistent with expected MSC biology (Fig. 3). Furthermore, assessment of cytokine production and immunophenotyping demonstrate that in the platform, immune cells switch from a pro-inflammatory to an anti-inflammatory state. Microvesicle size also changes in the presence of MSCs, though it is unclear if the increase in particle size is due to the fusion of vesicles or production of larger vesicles. Interestingly, while T cells, especially CD8 + T cells, were downregulated in the presence of MSCs, B cells were slightly upregulated. Indeed, the field is still unclear as to how MSCs affect B cells with studies showing contradicting data as to whether MSCs increase or decrease B cell proliferation^28,29^. It is possible that though they suppress total B cells, they may concurrently promote proliferation of B_regs_^30^. Future studies are to be conducted to characterize B cell subtypes in this bioreactor system.

We have previously found that system volume, time of exposure to MSCs and cross communication between MSC and T cells are important factors that affect MSC immunomodulation^22^. Consistently, here we found that different doses and duration of MSC exposure significantly modified their therapeutic effects. Results showed that though there was a dose response in regard to MSC number, increased perfusion time did not lead to more immunomodulation. In fact, in this system, the shorter time frame of MSC exposure (24 hours) led to the most significant anti-inflammatory effects (Fig. 6). This may in part be due to 1) the timing of the lymphocyte activation and the fact that unlike in an *in vivo* injury system, the source of inflammation is “static” and/or 2) the fact that monocytes, after a few days in culture will die off. Monocyte mediated MSC-suppression of T lymphocytes has been demonstrated in a study showing that blood monocytes activate MSCs via IL-1β which in turn inhibits T cells through their secretion of TGF-β1^31^. Here we show that monocytes play a broader role in MSC driven immunomodulation (Fig. 8). The involvement of monocytes in MSC driven immunomodulation has recently been given a lot of attention with studies showing that infused dead MSCs can still impart some therapeutic effect in animal models of liver injury^32^ and more recently in GvHD^33^. It is thought that this is due to monocytes engulfment of dead MSCs and their resulting development of an immunoregulatory phenotype^34^. The present study seems to suggest that even in the absence of dead MSCs, monocytes play an important role in lymphocyte changes induced by MSCs. More studies, including shorter duration studies, need to be developed to study this phenomenon as well as the effect of the MSC bioreactor on other immune populations, such as Th17 cells and Tregs, that have been previously described to be affected by MSCs^35^.

The potential for clinical utilization of this *ex vivo* MSC technology was a guiding factor in its design. While strong preclinical data exists, MSCs have been in long-standing clinical testing with limited therapeutic effects observed in humans^7,9,36–38^. This disparity is hypothesized, in part, to be directly related to how these cells are delivered. There is wide debate on the effects of prolonged *ex vivo* cell culture of MSCs^39^, whether their potency changes with cryopreservation^40–42^, and if infused systemically they get trapped in the lungs^13^ or induce an innate immune attack^18^. Our ex vivo MSC technology was designed to overcome these limitations by housing MSCs in an ex vivo blood-contacting device that significantly extends the duration of cellular therapeutic activity compared to injection. MSCs can, therefore, be administered in higher doses (i.e. number of cells) relative to injection or IV infusion without the risk for micro-embolisms associated with cell clumping during MSC injections. Beyond MSC survival, the technology allows for sustainable delivery of an MSC-derived combinatorial mixture of natural anti-inflammatory and trophic molecules to the blood.

Given its ex vivo design there are many applications in which this technology could be utilized. One embodiment is to integrate MSC bioreactors into a hemofiltration circuit or extracorporeal circuit such as dialysis, ECMO or apheresis to deliver MSC factors directly to the blood stream. This would preserve the dynamic response of the MSCs to the patient’s own inflammatory signals. This modality is undergoing human testing in patients with dialysis dependent acute kidney injury^43^, NCT03015623). In this severely ill patient population, the study is evaluating ex vivo MSC therapy as an approach to address the underlying systemic inflammatory processes while accelerating organ repair. Another way to deploy this technology is to condition a collection of a patient’s own blood, independent of a patient-connected blood circuit. This approach of autologous blood conditioning has potential applications for a variety of disease models, such as those characterized by systemic dysregulation of the peripheral immune cell populations. Without the need for continuous vascular access, this embodiment renders ex vivo MSC therapy more amenable for chronic diseases where multiple interventions may be needed. With a unique perspective on compartmental control and analysis of MSC activity, further testing will determine whether ex vivo MSC bioreactors can be translated directly as a combination product that continuously reprograms a patient’s immune system and/or as a platform that can be applied clinically to autologous blood products.

## Materials and Methods

### Stem cell donors

Mesenchymal stromal cells (MSC) were isolated from human bone marrow aspirate (n = 1 23 y/o female donor) obtained from Lonza (Lonza, MD, USA) and cryopreserved at early passage (P3-P5). All human samples were obtained by commercial vendors under a consented protocol for research purposes only.

### Media preparation

MSC seeding media consisted of aMEM supplemented with 2.5% human serum albumin (HSA; Grifols, Barcelona, Spain). Media used for circuit perfusion (without PBMCs) consisted of aMEM supplemented with 10% fetal bovine serum (FBS, GE Healthcare). Media used for PBMC circuit perfusion and flask culturing was comprised of RPMI 1640 media supplemented with 10% FBS (GE Healthcare), 1% antibiotic-antimycotic, and 0.2% gentamicin (Sigma-Aldrich) was used as culture medium.

### Microreactor MSC seeding

MSCs thawed from cryopreservation into aMEM supplemented with 2.5% HSA, counted via Trypan Blue exclusion. The desired cell number was suspended into 9 mL of aMEM and then seeded into saline-primed microreactors (Spectrum Laboratories, CA, USA; C02-P20U-05) with 0, 1, 3, 6, or 9 ×10^6^ viable cells per device. Excess media flowed through while cells remain within the extraluminal space of the reactor. Microreactors used were 20 cm long an internal surface area of 28 cm^2^. Cell densities within the reactor ranged from 35,714 cells/cm^2^ for the 1 M seeded MR to 321,428 cells/cm^2^ for the 9 M seeded MR. Within each microreactor are nine 0.5 mm diameter fibers comprised of polyethersulfone with a 0.2 μm pore size. The total internal volume of the microreactor is 1.5 mL.

Microreactors were then placed into the 37 °C for 2 hours to allow for cell attachment and were subsequently held between 4-24 hours at room temperature prior to integration into the circuit. Hold times cover the range of expected delivery of the device from cell seeding location to clinical application.

### PBMC isolation and enrichment

Peripheral blood mononuclear cells (PBMCs) obtained from AllCells LLC (AllCells, CA, USA) were freshly isolated by density gradient centrifugation using Ficoll-paque (GE Healthcare). CD14 + Monocytes were enriched by CD14 MicroBeads (Miltenyi Biotech, Bergisch Gladbach, Germany) according to the manufacturer’s instructions. Informed consent was obtained for each donor by individual vendors according to vendor‐specific protocols and institutional review board review. All human samples were obtained by commercial vendors under a consented protocol for research purposes only.

### Proliferation ViaFluor labeling

A ViaFluor 488 (FITC) stock (5 mM in DMSO; Biotin, CA, USA) stored at -20 °C, was thawed and diluted in phosphate-buffered saline (PBS) to the desired working concentrations. Freshly isolated PBMCs were resuspended in PBS at 3 ×10^6^ cells per mL and incubated with ViaFluor (final concentration: 1uM) for 15 min at room temperature. Cells were washed and resuspended in culture medium for 25 min at 37 °C to stabilize the ViaFluor labeling. After a final wash step, cells were resuspended in culture medium at 2.7 ×10^6^ cells per mL then activated with PHA (5 ug/mL) and IL-2 (100 ng/mL) (all Sigma-Aldrich).

### Flow cytometric analysis

All monoclonal antibodies were obtained from BioLegend (San Diego, CA, USA). Antibodies specific for human PBMC surface markers were: Panel 1 CD4-Per-CP-Cy5.5, CD8a-APC-Cy7, CD19-PE, CD25-APC, CD38-PE-Cy7 and CD69-PerCP-Cy5.5; Panel 2 CD4-Per-CP-Cy5, CD183/CXCR3 (Th1 marker) and CD194/CCR4 (Th2 marker); Panel 3 HLA-DR-PE-Cy7, CD14-APC, CD16-APC-Cy7, and CD69-PerCP-Cy5.5.

Staining was done in a total volume of 100 uL Stain Buffer containing FBS and ≤0.09% sodium azide (BD Biosciences). PBMCs were recovered and incubated for 30 min at 4 °C with fluorophore conjugated mAbs then analyzed on a FACSCanto II flow cytometer (BD Biosciences) using BD FACSDiva v6.1.1 software. Viable cells were gated based on forward/side scatter. Single stain composition was set using Invitrogen UltraComp eBeads (ThermoFisher) and used to gate total and activated T cell populations. Flow cytometry analysis was performed in FlowJo (FlowJo LCC, OR, USA; version 8.7.3). Proliferative cells (cells labeled with ViaFluor 488; FITC) gates were set using a non-stimulated static control (no PHA/IL-2).

### Analysis of cytokine production

Cell supernatants were collected and frozen down at -80 °C until assayed using a Human 42 Multiplex Discovery Assay with Luminex xMAP technology (EGF, Eotaxin-1, FGF-2, Flt-3L, Fractalkine, G-CSF, GM-CSF, GROα, IFNα2, IFNγ, IL-1α, IL-1β, IL-1ra, IL-2, IL-3, IL-4, IL-5, IL-6, IL-7, IL-8, IL-9, IL-10, IL-12 (p40), IL-12 (p70), IL-13, IL-15, IL-17A, IL-18, IP-10, MCP-1, MCP-3, MDC, MIP-1α, MIP-1β, PDGF-AA, PDGF-AB/BB, RANTES, sCD40L, TGFα, TNFα, TNFβ, VEGF-A; Eve Technologies, AB, Canada). The level of VEGF, IL-6, and PGE2 in cell culture supernatants were also evaluated using ELISA (Human DuoSet ELISA Development Kits (VEGF, IL-6, PGE2), R&D Systems) with absorbance measured at 450 nm/570 nm.

### Measurement of metabolite production

Cell supernatants were collected and frozen down at -80 °C until analyzed using a Cedex Bio Analyzer (Roche Diagnostics, Basel, Switzerland), according to manufacturer’s instructions, in order to determine the rate of MSC metabolism of Glucose, Glutamine, and Pyruvate, and the production rate of Lactate, Ammonia (NH_3_), and Lactate Dehydrogenase (LDH).

### Longitudinal media-only perfusion of microreactors with day 9 stimulation

Thawed MSCs were seeded into saline-primed microreactors at 0, 1, 3, 6, and 9 ×10^6^ viable cells per device (n = 3). Microreactors were rinsed fresh media and incubated at 37 °C for 2 hours to allow for cell attachment prior to a 24 hour hold at room temperature as described previously.

Supplemented aMEM media (50 mL) was added to 15 tissue culture reservoirs (Origen, TX, USA), which were attached to circuit tubing (Cole Parmer, IL, USA; GH-96114-16). After 24 hours of room temperature hold, microreactors were integrated proximal to the reservoir, and perfusion was performed using a Masterflex pump (Cole Parmer) at flow rate of 10 mL/min perfused in 37 °C, 5% CO2, 95% Rh. Circuit sampling was performed at T = 0, 0.5, 4, 17, 24, 48, 72, 96, 144, and 196 hours of perfusion using a four-way, large-bore stopcock (Baxter).

At 8 days of perfusion (T = 196 hours), a solution of fresh stimulated supplemented aMEM media containing 10 ng/mL IFNγ, 1 ng/mL IL-1β, and 5 ng/mL TNFα was prepared at 35x concentration, and 1 mL was added to each stimulated perfusion circuit. Unstimulated circuits received 1 mL of fresh supplemented aMEM to serve as control. Circuits were sampled at T = 0.5 and 20 hours post-stimulation. All samples were frozen and kept at -80 °C until sample analysis was performed.

### Stimulated PBMC perfusion

Saline-primed microreactors were seeded with 0, 3, or 9 ×10^6^ viable MSCs per device as described previously, incubated at 37 °C for 2 hours to allow for cell attachment, and held for 4 hours at room temperature.

Viafluor labeled PBMCs acquired from AllCells (Quincy, Massachusetts) were resuspended at 2.7 ×10^6^ cells per mL in supplemented RPMI 1640 media then stimulated with 5 ug/mL PHA and 100 ng/mL IL-2 (all Sigma-Aldrich). Stimulated labeled PBMCs were then added to microreactor circuits (5 mL suspension per circuit) and perfused at flow rate of 1 mL/min at 37 °C, 5% CO2, 95% Rh, for 5 days.

Following perfusion, suspension was harvested by centrifugation, supernatant was frozen and kept at -80 °C until sample analysis was performed while cellular pellet was resuspended in 300 uL of Stain buffer, and immediately evaluated using Panel 1 described above by flow cytometry using BD FACSCanto II (BD Biosciences).

### MSC reprogramming of lymphocytes and monocytes

To examine whether MSC treatment had a durable effect on lymphocyte function, PHA/IL-2 activated PBMCs were perfused over 5 days in microreactor-integrated circuits (0, 3, and 9 ×10^6^ MSCs), as previously described. After 5 days, lymphocytes were harvested by centrifugation, resuspended in supplemented RPMI 1640 (900 uL), and cultured in T25 culture flasks (Corning) under static conditions for 2 days. Supernatant was collected and frozen at -80 °C until multiplex analysis to evaluate lymphocyte secretome. Cell pellet was stained with CD183/CXCR3 (Th1 marker) and CD194/CCR4 (Th2 marker) antibodies and assayed via flow cytometry.

To examine monocyte reprogramming, supernatant containing secreted mediators from a 5-day MSC-PBMC perfusion was washed onto monocyte cultures in a cell-repellent surface tissue culture dish (Greiner Bio-One, Cat #662970) for 2 days. After two days, culture was harvested by centrifugation, supernatant was frozen and kept at -80 °C until sample analysis, then resuspended in 300 uL of Stain buffer, and evaluated using Panel 3 described above by flow cytometry using BD FACSCanto II (BD Biosciences). Dead cells were excluded using Propidium Iodide staining (Fisher Scientific) following manufacturer’s instructions.

To assess the effect of monocytes in PBMC-MSC bioreactor, PBMCs were stimulated with PHA/IL-2 then cultured in T25 static culture flasks. At day 2 and day 4 of culture, purified monocytes were replenished in the T25 flask (~20% of PBMC). After replenishing monocytes at day 4, PBMC suspensions were perfused in prepared microreactor circuits (0 and 9 ×10^6^ MSCs) for 24 hours. After 4 days static and 24 hours perfusion, culture was harvested, supernatant was frozen, evaluated using Panel 1 described above by flow cytometry.

### Dose-duration peripheral blood mononuclear cell perfusion

On Day 0 microreactors were seeded with 0, 3, or 9 ×10^6^ viable MSCs, incubated, and held as described previously.

To measure the effect of MSC treatment on T cell proliferation, ViaFluor-labeled stimulated (PHA/IL-2) PBMCs were resuspended to 3.0 ×10^6^ cells per mL in supplemented RPMI 1640 media. PBMC suspension was then added to a T25 culture flask (Corning) and held at 37 °C for 24 hours. Cell suspension (5 mL per circuit) was then perfused in prepared microreactor circuits (0, 3, 9 ×10^6^ MSCs) at flow rate of 1 mL/min at 37 °C, 5% CO_2_, 95% Rh, for either 24 or 72 hours. Following perfusion, the cells were transferred to T25 culture flasks and incubated until reaching the Day 5 timepoint where the culture was harvested, supernatant was frozen, and evaluated using Panel 1 described above by flow cytometry.

### Measurement of extracellular vesicles (EV)

EVs isolated from cell culture supernatant were centrifuged at high force and analyzed using ViewSizer 3000 (Manta Instruments) with individual, simultaneous particle analysis ranging in size from 10 nm to 2 µm. The median diameter of EVs is presented in basal and activated MSCs as insets.

### miRNA analysis

Dynamic cultures were performed using a magnetically actuated perfusion pump (PURALEV 200MU, Levitronix, Framingham, MA). Medium used for all culture was a basal RPMI 1640 (Sigma, St Louis, MO) with 10% exosome free, heat-inactivated FBS. Exosome free FBS was produced by ultracentrifuging FBS at 100,000 g (L8-70M, Beckman, Brea, CA) for 18 hours and collecting resultant supernatant.

MSCs were allowed to adhere to hollow fiber bioreactor fibers overnight. Control groups (MSC, PBMC, and co-culture) were run with base media supplemented with 50 ng/mL of IL-2 (Sigma). Stimulated groups (PBMC and co-culture) were run with base media supplemented with 5 ug/mL PHA-L (Sigma) and 50 ng/mL IL-2 (Sigma). Stimulated MSCs were run with base media supplemented with 10 ng/mL each of IFNγ (R&D Systems, Minneapolis, MN), TNFα, IL-1β, and IL-17 and 50 ng/mL IL-2 (Sigma). All experiments were run for 96 hours.

PBMCs were harvested at the end of each experiment, pelleted, and frozen in a 90% FBS/10% DMSO freezing solution. MSC culture media was collected and processed in the same way to produce exosome free FBS except that the supernatant was instead collected and the remaining faint pellet was resuspended in 1 mL of supernatant (the supernatant was then discarded) and frozen down at -80C until further analysis.

miRNA multiplex analysis was performed using the FirePlex miRNA assay (Abcam, Cambridge, MA). Group normalization was performed using the FirePlex Analysis Workbench (Abcam).

### Exosome staining

Ultracentrifuged material from stimulated MSC cultures was assessed for exosome/miRNA presence using the SYTO RNASelect Green Fluorescent Cell Stain per vendor provided instructions (Thermo Fisher Scientific, Waltham MA). Processed material was mixed 1:1 with exosome free RPMI media and subsequently cultured with fresh from frozen PBMCs for 24 hours. Imaging was performed using an EVOS Imaging System (Thermo).

### Statistical analysis

Evaluation for ELISA, multiplex bead-based assay, proliferation and immunophenotypic results was performed in Prism (GraphPad Software, La Jolla, CA). Individual tests are described in figure legends depending on the relevant comparative analysis performed for each study. Results are presented as mean ± standard deviation. Two-factor analysis of variance (two-way ANOVA) and un-paired student t-tests were used to compare differences among groups depending on the study. Values of p < 0.05 were considered statistically significant for all analyses.

## Supplementary information

Supplementary information.
