# Author Correction: Mesenchymal Stromal Cell Bioreactor for *Ex Vivo* Reprogramming of Human Immune Cells

**DOI:** 10.1038/s41598-020-72947-y

**Published:** 2020-09-17

**Authors:** Ashley Allen, Natalie Vaninov, Matthew Li, Sunny Nguyen, Maneet Singh, Peter Igo, Arno W. Tilles, Brian O’Rourke, Brian L. K. Miller, Biju Parekkadan, Rita N. Barcia

**Affiliations:** 1 Sentien Biotechnologies, Inc., Lexington, MA 02421 USA; 2 Department of Surgery, Center for Surgery, Innovation, and Bioengineering, Massachusetts General Hospital, Harvard Medical School and Shriners Hospitals for Children, Boston, Massachusetts 02114 USA; 3 Harvard Stem Cell Institute, Cambridge, Massachusetts 02138 USA; 4 Department of Biomedical Engineering, Rutgers University, Piscataway, New Jersey 08854 USA

Correction to: Scientific Reports https://doi.org/10.1038/s41598-020-67039-w, published online 23 June 2020

Maneet Singh was omitted from the author list in the original version of this Article. This has now been corrected in the PDF and HTML versions of the Article, and in the accompanying Supplementary Information file.

The Author Contribution section now reads:

“Conceptualization, R.N.B., B.P. Execution of Experiments, A.A., M.L., N.V., S.N., M.S., B.O.R.; Data Analysis and Review, A.A., M.L., N.V., S.N., M.S., P.I., B.O.R., B.P., R.N.B., A.T., B.L.K.M.; Manuscript Preparation, A.A., M.L., N.V., S.N., M.S., P.I., B.O.R., B.P., R.N.B.; Funding Acquisition, B.P., B.M.”

In addition, the original Acknowledgements statement contained information that should have been given in the Competing Interests section. This Competing Interests section has been updated to include information about the omitted author and now appears in the Article as below:

“A.A., N.V., S.N., P.I., A.T., B.O.R., B.L.K.M. and R.N.B. are employees and equity shareholders of Sentien Biotechnologies. B.P. and B.L.K.M. are also equity shareholders and B.P. is an inventor of the technology with licensed patents to Sentien for commercialization. M.S. is a former employee of Sentien Biotechnologies and is at present an employee at Mitobridge, Inc. with no other competing financial interests. M.L. is an inventor of this technology and at present time an employee of Vor BioPharmaceuticals with no other competing financial interests.”

Finally, the Acknowledgements,

“A.A., N.V., S.N., P.I., A.T., B.O.R., B.L.K.M. and R.N.B. are employees and equity shareholders of Sentien Biotechnologies. B.P. and B.L.K.M. are also equity shareholders and B.P. is an inventor of the technology with licensed patents to Sentien for commercialization. M.L. is an inventor of this technology and at present time an employee of Vor BioPharmaceuticals with no other competing financial interests. This research was conducted with private funding and support under SBIR Grant No. R44DK085766 and R44HL128659 awarded by the National Institutes of Health as well as R01EB012521 and R21AI134116.”

now reads:

“This research was conducted with private funding and support under SBIR Grant No. R44DK085766 and R44HL128659 awarded by the National Institutes of Health as well as R01EB012521 and R21AI134116.”

