# Supplementary material for: Mesenchymal Stromal Cell Bioreactor for Ex Vivo Reprogramming of Human Immune Cells
Source: Sci Rep. 2020 Jun 23;10:10142. doi: 10.1038/s41598-020-67039-w (PMC7311545; doi:10.1038/s41598-020-67039-w)

Supplementary Information

Mesenchymal Stromal Cell Bioreactor for Ex Vivo Reprogramming of Human Immune Cells

Ashley Allen[1+], Natalie Vaninov[1+], Matthew Li[2], Sunny Nguyen[1], Peter Igo[1], Arno W. Tilles[1], Brian O'Rourke[1], Brian L. K. Miller[1], Biju Parekkadan[1,2,3,4], Rita N. Barcia[1]*

[1] Sentien Biotechnologies, Inc., Lexington, MA 02421 USA
[2] Department of Surgery, Center for Surgery, Innovation, and Bioengineering, Massachusetts General Hospital, Harvard Medical School and Shriners Hospitals for Children, Boston, Massachusetts 02114, USA
[3] Harvard Stem Cell Institute, Cambridge, Massachusetts 02138, USA
[4] Department of Biomedical Engineering, Rutgers University, Piscataway, New Jersey 08854, USA

[+]Equal contribution
*Corresponding Author

Ashley Allen- ms.ashal@gmail.com
Natalie Vaninov- Natalie.Vaninov@gmail.com
Matthew Li- Li.Matthew14@gmail.com
Sunny Nguyen- Sunny.Nguyen@sentienbiotech.com
Peter Igo- Peter- Igo@sentienbiotech.com
Arno W. Tilles- Arno.Tilles@sentienbiotech.com
Brian O'Rourke- Brian.ORourke@sentienbiotech.com
Brian L.K. Miller- Brian.Miller@sentienbiotech.com
Biju Parekkadan- Biju.Parekkadan@sentienbiotech.com
Rita Barcia- Rita.Barcia@sentienbiotech.com

Figure S1

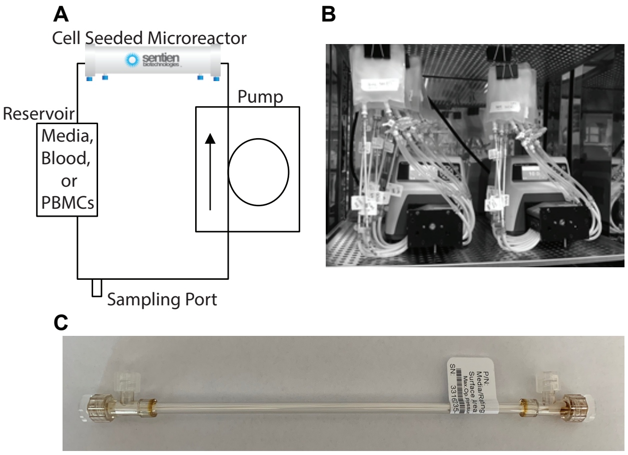
A
B
C
Cell Seeded Microreactor
sentien
Reservoir
Media,
Blood,
or
PBMCs
Pump
Sampling Port

Figure S2

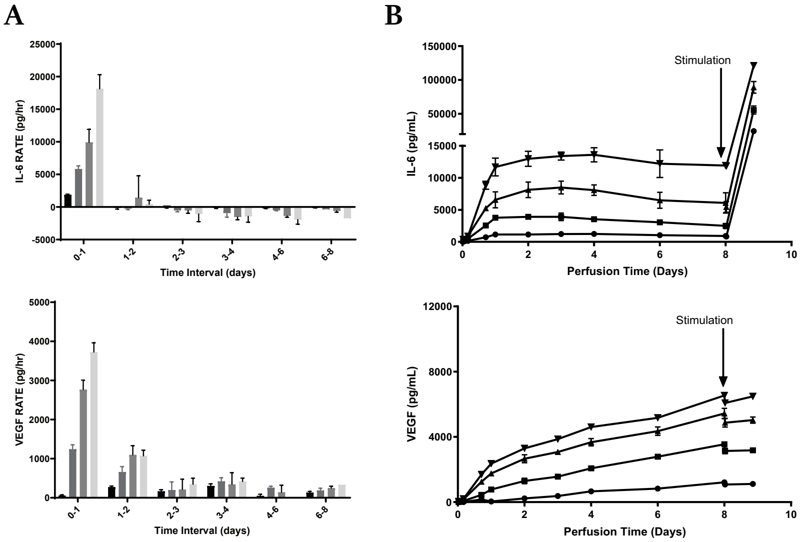
A
B
IL-6 RATE (pg/hr)
Time Interval (days)
0-1
1-2
2-3
3-4
4-6
6-8
VEGF RATE (pg/hr)
IL-6 (pg/mL)
Perfusion Time (Days)
Stimulation
VEGF (pg/mL)

Figure S3

A

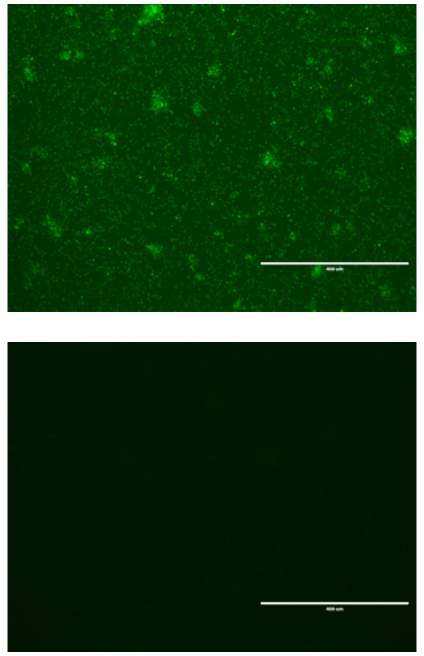

MSC UC + PBMC

MSC Sup + PBMC

B

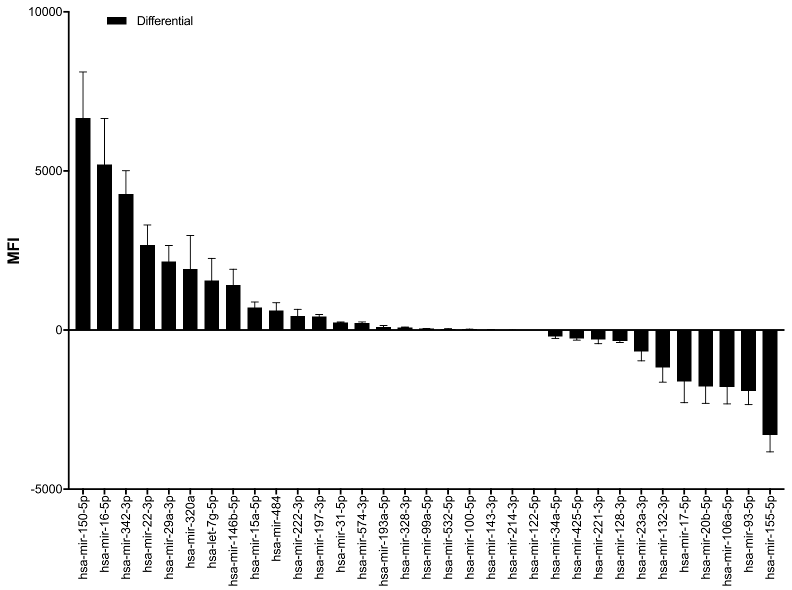

Supplement: Supplementary file 1 — Supplementary information. [file 41598_2020_67039_MOESM1_ESM.pdf]
